# “Simply complicated”: Uncovering the processes of lifestyle behavior change among college and university students with access to a digital multiple lifestyle intervention

**DOI:** 10.1177/20552076241245905

**Published:** 2024-04-09

**Authors:** Katarina Åsberg, Ann Catrine Eldh, Marie Löf, Marcus Bendtsen

**Affiliations:** 1 Department of Health, Medicine and Caring Sciences, 4566 Linköping University, Linköping, Sweden; 2 Department of Public Health and Caring Sciences, Uppsala University, Uppsala, Sweden; 3 Department of Biosciences and Nutrition, 27106 Karolinska Institute, Stockholm, Sweden

**Keywords:** Behavior change, lifestyle, mHealth, psychology, qualitative, studies, alcohol, diet, physical activity, smoking, public health, health-related behavior change, college and university students

## Abstract

**Background:**

One approach to promoting healthy lifestyle behaviors is to target students with digital interventions. One of these is the digital intervention Buddy. This study aimed to understand why college and university students’ chose to participate in a digital multiple lifestyle behavior intervention trial (Buddy), and their subsequent experiences of the behavior-change process.

**Methods:**

College and university students taking part in a trial of the Buddy intervention were individually interviewed after completing the 4-month intervention. Participants were guided to narrate their experiences and actions that followed signing up. Altogether, 50 interviews were conducted via telephone. The verbatim transcribed texts were analyzed qualitatively.

**Results:**

The analysis generated seven personas, which illustrated the students’ different levels of engagement with the intervention and the behavior-change process. These were: the Occupied, the Kickstarter, the Aimless, the Reflective, the Goal-oriented, the Compliant, and the Personally developed. Buddy worked best for students who had clear ideas about what they wanted to change and why, and who were aware of their needs, and those who could translate information and reflection into action and had the mental and physical energy needed to make changes.

**Conclusions:**

The progress of behavior change depends on the interaction between the digital mode of delivery, the intervention materials of Buddy, the individual's expectations, needs, and skills, and their current life situation. This suggests that designing lifestyle interventions could benefit from more often considering the various personas’ different intentions, knowledge, and contexts. By doing so, interventions are likely to emerge that can better match different needs in the target population.

## Introduction

Unhealthy lifestyle behaviors, including physical inactivity, unhealthy diets, smoking, and harmful alcohol consumption, are leading causes of noncommunicable diseases (NCDs) globally, including cardiovascular disease and several types of cancers. Despite being modifiable risk factors, their prevalence remains high in both adult and young populations, placing avoidable burdens on society.^1,2^ Therefore, it is essential to understand how we can prevent unhealthy lifestyle behaviors from developing, and how we may best support those who can benefit from changing their behaviors. The global action plans for preventing and controlling NCDs^3^ and for promoting physical activity^4^ published by the World Health Organization emphasize that there are opportunities for prevention at multiple stages throughout life, and that digital interventions can be used to promote healthy lifestyles and support individuals in changing their behaviors.

Relying on behavior-change theories or techniques has been found to be important when developing digital interventions^5–8^; however, it is also important to recognize that target populations of public health interventions are typically heterogeneous. One way to conceptualize this heterogeneity in the development process is to use personas, or user profiles, which describe fictional persons who represent archetypes within the target population. In this study, we developed personas based on interviews with college and university students who had been given access to a digital intervention, with the aim to expand on what we know regarding how students’ experience the behavior-change process.

### Unhealthy lifestyle behaviors among college and university students

One opportunity to reduce the burden from unhealthy lifestyle behaviors is to promote healthy behaviors among college and university students. Transitions into and through higher education involve multiple concurrent changes for many students, including navigating new physical and social environments, learning to succeed academically, and taking on new financial and household responsibilities. Being a student often means living on a limited budget, sharing student accommodation, and participating in a social context involving a new culture that may sometimes normalize unhealthy lifestyle behaviors.^9,10^ But this transition could also work as a window of opportunity to intervene^11^ since students engage in various unhealthy lifestyle behaviors.^12–15^ Among 18- to 29-year-olds in Sweden who responded to a national public health survey (n = 1610), approximately one-third were physically active for <20 min per day or were sedentary for more than 10 h per day. In addition, nearly 13% smoked daily or occasionally and one in four had risky alcohol consumption, which at the time of the study was defined as either drinking 9 (women)/14 (men) or more standard drinks of alcohol per week or drinking 4 (women)/5 (men) or more standard drinks on a single occasion at least once a month (a standard drink is in Sweden defined as 12 g of pure alcohol (ethanol/ethyl alcohol),^16^ for example 125 mL of wine or half a pint of beer). Furthermore, students report high levels of reduced mental wellbeing due to stress, symptoms of exhaustion, anxiety, depression, and worries about being unable to cope with their studies.^17^ These unhealthy behaviors are concerning because unhealthy behaviors established during young adulthood may be sustained, which will have an adverse impact on health later in life.^18–20^ Conversely, *healthy* lifestyle behaviors support good overall physical and mental health in the everyday life of students,^21^ and have also been positively associated with modestly higher grade averages.^22^

### Digital interventions to support lifestyle behaviors

Efforts to reach students often utilize technological developments to interrupt trends in unhealthy lifestyle behaviors.^23–26^ Digital interventions, typically delivered through websites, text messages, apps, and virtual games, are an example of this, and have proven effective in improving many health behaviors.^6,27–29^ For instance, findings indicate that digital brief alcohol interventions produce results comparable to face-to-face interventions.^6^ Similarly, a systematic review concluded that a variety of approaches, both face-to-face and digital, are effective for tobacco cessation.^30^ Digital methods are also scalable and feasible for large populations, making them relevant in the field of public health. Previous evaluations of digital lifestyle interventions among college and university students have mainly focused on alcohol consumption, drugs, and tobacco use with promising results for smoking and alcohol interventions but mixed results for drug use.^31,32^ However, there are fewer evaluations of digital interventions promoting multiple health-related behaviors, encompassing alcohol, diet, physical activity, and smoking simultaneously.^33^

### The Buddy intervention

In a previous study, we investigated and found that students seek to balance their desire to make informed health choices against experiencing and enjoying student life.^34^ These challenges and needs of students that have been previously identified have informed the design and content of a digital multiple lifestyle behavior intervention called *Buddy*. Buddy aims to provide health behavior-change support regarding alcohol consumption, physical activity, diet, and smoking.^35^ The design of Buddy was based on previous research of digital lifestyle interventions among Swedish university students,^23–25,36–40^ on social cognitive models for behavior change,^41,42^ and on behavior-change techniques in the Behaviour Change Technique Taxonomy, version 1 (BCTTv1 93-item taxonomy).^43^ Buddy is a mobile phone based, fully automated intervention that is designed as a low-intensity intervention, which does not require participants to engage with the materials daily. Each Sunday afternoon, over a period of 4 months, participants receive an automated text message with a link and a reminder to access the support materials. The support materials focus on modifying participants’ environment, intentions, and skills—which are often highlighted as important factors for change according to social cognitive models.^41,42^ These three factors are also prominent in the COM-B model (Capability, Opportunity, Motivation-Behaviour model) which was developed by Michie et al.^44^ The COM-B model suggests that behavior change is made possible by an individual's knowledge and skill regarding how to change behavior (capability), external circumstances giving rise to opportunities for behavior-change (opportunity), and conscious decision-making and emotional responses (motivation). We identified and designed support material components based on these cornerstones of the COM-B and other social cognitive models, resulting in the following six components: (1) screening and feedback, (2) goal-setting and planning, (3) motivation, (4) skills and know-how, (5) mindfulness, and (6) self-authored text messages. A full description of the Buddy intervention is available in Supplemental Appendix A.

### Individual resources and the use of personas in behavior-change research

Developing interventions based on current knowledge regarding health behavior change is important, however, interventions also need to consider the resources that the individual users are already in possession of. For instance, Schroé et al.,^45^ showed that participants who dropped out of a digital physical activity intervention scored high on psychological determinants; action planning, coping planning, and self-monitoring scales. Thus, those who dropped out were already familiar with the resources provided for them and found no or limited additional value from the intervention. Other types of support were needed for these individuals, indicating the necessity to take into consideration the heterogeneity within any target group regarding the resources that they as individuals possess.

One way to conceptualize and visualize such heterogeneity is the use of personas during the development of behavior interventions. A persona, or user profile, is a description of a fictive person who represents an archetype within the target population. While their use could shed light on the heterogeneity within a target population, LeRouge et al.^46^ argue that the benefit of personas has received little attention in healthcare informatics research. A review of methods employed for human-centered eHealth development only identified five studies using personas (among the 160 included studies), but one only conducted within the field of lifestyle behavior change.^47^ Another example is derived from a qualitative study by Burgermaster et al.^48^ Utilizing a phenomenological and social constructivist approach, the study identified four psychosocial phenotypes of behavior change in response to a school-based childhood obesity prevention intervention. The results illustrate how different subgroups of young adults responded differently to the intervention, and authors emphasize that behavior change is not a one size fits all.

## Objectives

While there is a need for behavior-change support for university students, it is critical that any such support is both suitable and acceptable. There is a lack of studies investigating students’ experiences of the behavior-change process when given access to support targeting multiple behaviors at the same time. In addition, the match between intervention design and the resources students already possess is understudied. Therefore, this study aimed to understand why college and university students’ chose to participate in a trial of the Buddy intervention and their subsequent experiences of the behavior-change process.

## Methods

This qualitative study of students’ experiences of the behavior-change process when given access to a digital support tool was nested within a factorial effectiveness trial of the Buddy intervention. The trial targeted college and university students across Sweden.^35^ The study received ethical approval on 2020-12-15 (Dnr 2020-05496), and is part of the MoBILE research program (Mobile health multiple Behavior Interventions across the LifEspan).^49^ The setting was a total of 19 colleges and universities in Sweden (out of 31), with medical, philosophical, and technical programs at undergraduate, post graduate and PhD levels. Students at these universities were enrolled through the standard national recruitment procedures, and attending these universities does not require any educational fees. Recruitment for the trial was by: (1) paper advertising (posters and leaflets), (2) digital advertising (email, social media, and learning platforms), and (3) through student healthcare staff. Participants who were eligible to take part in the trial were Swedish speaking students (≥ 18 years) who had at least one unhealthy lifestyle behavior (harmful alcohol consumption, insufficient physical activity, poor diet, or smoking). All participants who completed the 4-month follow up were asked if they would be willing to take part in an interview about the support they had received, regardless of whether the students had used the intervention or decided to stop using it. This manuscript presents the findings from these interviews and includes applicable items from the Consolidated criteria for reporting qualitative research (COREQ).^50^ A complete COREQ checklist can be found in Supplemental Appendix B.

Between September 2021 and May 2022, 87 students agreed to be interviewed (according to their responses in the 4-month follow-up questionnaire). They were contacted via text message and telephone to arrange a time and date for their interview. The text message included a link to the written study information. We contacted students in chronological order. Recruitment of students continued until the data material indicated and exceeded a comprehensive understanding of phenomena and meaning saturation was reached (n = 50).^51^ All the interviews took place on the phone, over a secure link, and began with the interviewer verbally repeating the study information, including its voluntariness and the right to withdraw without any need to justify such a decision. To meet the Swedish research ethics standards, consent needs to be documented (either written, verbal, or digitally). In this study, due to the format of the data collection, we certified individual verbal consent, which was audio recorded before the interview commenced. The characteristics of the 50 participating students are presented in Table 1.

**Table 1.** Characteristics of interview participants.

Participants’ baseline characteristics	Total (n = 50)
Sex female, n (%)	35 (70%)
Age, mean (SD)	31.3 (6.4)
Body mass index (kg/m^2^) mean (SD)	25.9 (4.4)
*Alcohol*	
Total weekly alcohol consumption, mean (SD)	2.9 (4.9)
Frequency of heavy episodic drinking, mean (SD)	1.0 (1.9)
*Smoking*	
Number of smokers, n, (%)	6 (12%)
Number of cigarettes last week, mean (SD)	6.0 (22.4)
*Physical activity*	
Weekly moderate physical activity minutes, mean (SD)	134 (204)
Weekly vigorous physical activity minutes, mean (SD)	46 (93)
*Dietary behavior*	
Fruit consumption, portions per week, mean (SD)	0.6 (0.7)
Vegetable consumption, portions per week, mean (SD)	0.9 (0.9)
Soft drinks, cans per week, mean (SD)	1.7 (3.7)
Candy and snacks, portions per week, mean (SD)	6.0 (5.7)

### Data collection

The first author, who had no prior or current relation with the respondents, conducted all the interviews using a study-specific, semistructured interview guide consisting of open-ended questions (Supplemental Appendix C). The first author has a background in public health and previous experience in conducting qualitative research, supported by an expert in the research team providing guidance. The interviews were conducted without presence of nonparticipants and no field notes were made. The interview guide was influenced by a phenomenological notion where the intention was for each participant to focus on their lived experience, considering the individuals’ thoughts and conscious outlooks. This gives the researchers access to the essence of a shared understanding.^52^ Consequently, the interview guide invited respondents to reflect upon their individual process, from coming into contact with the trial and the intervention, and the events that followed: First, respondents were asked about the moment when they had first come into contact with the intervention, and their thoughts about health and lifestyle at that time. Next the respondents were asked to reflect upon their experiences when accessing the intervention, and whether they had made any lifestyle behavior changes over the period that followed. Lastly the interview was concluded by asking about respondents’ experiences and understanding of behavior change in general. The interviews lasted for a mean time of 20 min (range 4–32 min). Although all questions in the interview guide had been exhausted within this range, there was no set maximum time for the interviews. All the interviews were audio recorded and transcribed verbatim by a skilled secretarial service, resulting in 413 pages of single-spaced text. Prior to the analysis, all the interview transcripts were checked for accuracy against the audio files.

### Data analysis

The qualitative data analysis focused on the respondents’ narratives and their experiences of the behavior-change process. This was conceptualized as “what happened” (described at any given point) and the individual's reconstruction and understanding of that experience. The analysis was influenced by the methodological steps outlined in Lindseth and Norberg's^53,54^ phenomenological hermeneutical method including: (i) naïve reading, (ii) structural analysis, and (iii) comprehensive understanding. Initially, in order to construct an overview of all 50 respondents’ narratives, each interview was read and formulated as a short text recapturing its essence. Next, all of these 50 naïve understandings were read separately and then jointly, forming one raw comprehension of the entire dataset. Through this process, patterns regarding similarities and commonalities transpired across participants and seven personas (the Occupied, the Kickstarter, the Aimless, the Reflective, the Goal-oriented, the Compliant, and the Personally developed) were formed. These discrete personas—equating to “voices”—represent archetypical respondents in terms of their intentions and experiences of the behavior-change process. To portray the experiences of these seven personas, meaning units associated with each of them were marked (using NVivo 12 Plus and open coding), generating a total of 531 meaning units in accordance with the qualitative content analysis process suggested by Elo and Kyngäs.^55^ During this process, we explored whether there was any overlap between personas, or whether personas were missing. We found that we could neither merge personas with each other without losing granularity and nuance of findings nor add new personas without creating overlap. To finalize the structured analysis, individual rounds were conducted with the verbatim transcripts, identifying meaning units common among all the respondents regarding their behavior-change processes. A total of 278 meaning units were generated, revealing commonalities among the personas. To conclude, a comprehensive understanding of students’ reasons to participate, experiences of, and learning through the Buddy intervention was formed. The first author (KÅ) conducted the analysis with the second (ACE) and last (MB) co-authors reading and discussing all the steps of the analyses, including how codes were extracted and defined, the forming of the seven personas, and the emerging and concluding findings.

## Results

The seven personas representing respondents’ intentions and experiences of the behavior-change process are described in this section, in active voice. An overview of the seven personas, their alias, a consensus description for each, and the proportion of interviews that built the personas can be found in Table 2. Each persona was given an alias and a one-sentence description in order to easily distinguish them from one another. The results section concludes with a presentation of our comprehensive understanding of the findings. The consensus voice of the personas was created by the authors, shaped by means of the analysis with neither quotes nor raw extracts.

**Table 2.** Persona alias, consensus voice, and proportion of interviews that built each persona.

Persona alias	Description	n (%)^a^
The Occupied	Buddy didn’t help me, so I continued as before	7 (14)
The Kickstarter	Buddy helped me initially, but then I lost interest	4 (8)
The Aimless	I changed due to external circumstances	6 (12)
The Reflective	Buddy made me reflect – but not necessarily change	11 (22)
The Goal-oriented	Buddy didn’t help me, so I sought help elsewhere	7 (14)
The Compliant	Buddy helped me change	9 (18)
The Personally Developed	I was already making changes	6 (12)

^a^ Number and percent of interviews that built the persona.

### The seven personas

***The Occupied:*** Buddy didn’t help me, so I continued as before.

Digital support like Buddy has to provide me as a user with considerable stimulation; otherwise, I quickly lose interest. The invitation to try out Buddy made me curious, and it felt worth trying because there are always things one can change to be healthier. However, Buddy didn’t inspire or motivate me, nor did it provide any new information. I expected more from Buddy, including feedback, encouragement, and practical approaches. I’ve tried but I’ve been unsuccessful in changing my behavior due to a lack of mental strength, time, and energy. Many other things are important in my life at the moment: my studies, work, and family, and I can’t prioritize my health. I have too many commitments, which is not a sustainable strategy in the long run, but I need more time and energy to be able to exert myself further. At the same time, I’m unsure if I need to change—I found that the feedback from Buddy confirmed that I have relatively healthy behaviors after all.

***The Kickstarter:*** Buddy helped me initially, but then I lost interest.

I signed up to the Buddy trial because I’d been thinking about changing my lifestyle. I hoped to benefit from it and thought it could be an opportunity to gain some motivation and discipline. Motivating yourself can be easier when someone is observing your accomplishments. Initially, Buddy supported me mentally and encouraged me to choose different actions than before. For example, when the first text message arrived, I actively chose to ride my bike instead of using the car, I included more vegetables in my meals, and took morning walks. But I lost both interest and motivation after a few weeks and started to ignore the text messages. Looking back at it, I realize that I expected Buddy to be more interactive, varied, and customized based on my preferences for change and the actions I took. For example, I don’t drink soda, so that was not an issue, making receiving information on that topic unnecessary. I would have enjoyed more adjustments following my personal change in order to remain motivated.

***The Aimless***: I changed due to external circumstances.

I’m not sure why I signed up. I got curious and wanted to see what new service was available. I’d hoped that Buddy could be a push in the right direction, but I didn’t use it very much. It wasn’t what I’d expected; I found it too repetitive, and so I lost interest, and it didn’t help me change. When asked to report my behaviors at the end of the week, I found it difficult to recall what those behaviors had looked like. I also found it difficult to write motivating messages to myself since my preferences change from day to day. Out of curiosity, I picked some behavior-change challenges in Buddy that were irrelevant to me, making the reminders I got about these challenges rather pointless. I have made changes, but that had nothing to do with the support I received through Buddy. It was all due to external circumstances, such as moving, getting pregnant, the COVID-19 situation getting better, a calmer study period, economic and social situations, and the sunnier season. So, there have been changes, but I haven’t actively done anything to initiate those changes.

***The Reflective***: Buddy made me reflect—but not necessarily change.

I wasn’t sure what I wanted to change, but I wanted to see if Buddy could provide something positive for me. It turned out that Buddy reminded me of issues I would otherwise have easily down-prioritized on stressful days. Reporting behaviors at the end of the week became a meaningful routine for me, but I only did this when I was reminded to do so. The color-graded feedback was easy to understand, and it was satisfying to get a *green light*—a confirmation that you’d done something good for yourself during the past week. I was surprised that sometimes it took less effort than I’d thought to get a green light. But achieving the recommendations for fruits and vegetables was difficult, and I was red on alcohol. It was annoying to get a red light on the behaviors I had no intention of changing. I tried reaching the healthy lifestyle recommendations; for example, I had the alcohol consumption recommendations in mind every time I drank alcohol and tried not to exceed them. I also stood in the grocery store thinking about Buddy recommending healthy products, but I quickly forgot about all of it and acted the way I typically do. I do believe that awareness can help people change behaviors, and with Buddy I did become aware that I ate too few fruits and vegetables, ate too much candy and snacks, and drank too much soda and alcohol.

***The Goal-oriented***: Buddy didn’t help me, so I sought help elsewhere.

I felt a need to change and it seemed that signing up to the Buddy trial was as an opportunity to receive support. I know from past experience that unhealthy lifestyle behaviors have negatively affected my mental wellbeing, energy, and stamina. But Buddy didn’t match up to my expectations. It didn’t help me progress or succeed with the changes I wanted to achieve. The end-of-week reporting of behaviors didn’t take much time, but equally, it didn’t give much reflection either. It didn’t take long for me to start ignoring the end-of-week reporting when I realized that the content in Buddy stayed the same over time. What's more, I didn’t feel that I received the support I’d hoped for—I want more hands-on support and guidance tailored to my situation and needs. For example, I needed advice on how to bring about changes in practice. It turned out that Buddy asked a lot from me as a user, requiring me to become more involved in the process of change. In this way, Buddy didn’t support me but was instead a catalyst, pushing me to seek advice and help from other sources that had previously supported me.

***The Compliant***: Buddy helped me change.

I knew I wanted to change and what I wanted to change, so the invitation to try out Buddy was timely. Although I had considered making changes before, I’d never gotten around to doing so. Before I’d fully committed to making a change, the reminders from Buddy were very annoying, but then I realized they were necessary to give me the push I needed. Some weeks I ignored the text messages and just wanted to forget the whole thing. But other times I felt like I could achieve the change I wanted. The reminders helped me most when they arrived at times when my motivation was low, and they reminded me to use coping strategies other than unhealthy ones. The end-of-week reporting was both challenging and insightful, making me feel accountable for my choices. I have made changes—I eat and sleep better and do more regular physical activity; I’m now smoke- and alcohol-free and I’ve gained a friendlier attitude toward myself. Buddy made me feel like I could manage the behavior change while simultaneously having other commitments, and I believe awareness is the key to change, which the tips in Buddy helped me with. A few seconds of reading made me reflect and be prepared to stay one step ahead of falling into old habits. But Buddy does put high demands on the user and assumes that they’re willing to confront themselves, have experience of trying different strategies, and have already come a long way on their journey towards change.

***The Personally developed***: I was already making changes.

I was actively making changes to help myself feel better when the invitation to try out Buddy turned up. In the past, I tended to do what others did, which often resulted in me quickly reverting to old behaviors because I felt that the changes didn’t suit me. Nowadays, I know why I want to change; I can see the goal and the value in doing so—healthy behaviors will help me avoid ill health, improve my quality of life, and help me reach my life goals. I’ve previously received counseling, which has helped me to better understand my behaviors and needs. Even if one falls back into old behaviors, the value of the work one puts into changing doesn’t go away, and I’ve decided to stop comparing myself to others and instead set realistic expectations for myself. I used Buddy to create routines and structure. Buddy taught me to always have fruits and vegetables at home, and the recommendations from Buddy stuck in my head, making me reflect daily; for example, on how much fruit or vegetables I ate. Reporting my behaviors at the end of the week became a routine of reflection, and the feedback I received was helpful. Buddy confirmed and reminded me that I was doing good things for myself and gave me the feeling that someone cared, even though I knew it was an automated program.

### Comprehensive understanding

Buddy sparked the students’ interest, and their participation in the trial was an opportunity to access a new form of support that could benefit them without any apparent disadvantages. Simultaneously, the students saw their participation as contributing to research, indicating that an altruistic motive was part of the motivation to sign up for the study. Some students signed up due to curiosity, without any explicit expectations or a strong desire to change, thinking it would be a bonus if anything positive came out of their participation. Meanwhile, others were more opportunistic and sought support to help them make changes. Among the opportunistic, some had previously thought about making changes, while others had previously made changes but fallen back into old habits. Yet again, other students had already started to make changes and expressed a clear desire for change in themselves. These were more specific about what they wanted to achieve and about their intentions during their participation.

Buddy worked best for the students with clear intentions about what they wanted to change and why, and who were aware of their own needs. In addition, those who were able to translate the intervention's information and reflections into action and had the mental and physical energy needed to make their change, were also the ones who most consistently described being supported by Buddy. These students were aware of their goals and needs but also said that they had found ways to utilize helpful support for themselves, regardless of whether this was Buddy or other resources. Their motivation for change was increased when there was an expectation of a substantial positive outcome, or when previous health consequences could be clearly linked to current health-related behaviors.

While a key concept in Buddy is the suggested self-assessment of behaviors, the prompts that the intervention sends to students to self-assess their behaviors only sometimes led to reflection as intended. Rather, students’ perception of themselves as healthy affected whether they considered the feedback from Buddy to be relevant, in spite of what the intervention may have suggested. In reality, what was going on in their social surroundings was more important and influential than the Buddy intervention, and this was most often the case for those students who were less engaged with the support they received.

## Discussion

This study aimed to understand why college and university students’ chose to participate in a digital multiple lifestyle behavior intervention trial, and their subsequent experiences of the behavior-change process. The analysis established seven personas, which illustrate students’ different levels of engagement with the intervention and the behavior-change process—although it did not result in lifestyle changes for all students. In addition, the results show that the progress of behavior change depends on interaction. That is, interaction between the intervention materials, the individual's expectations, needs, skills, and their current life situation. This suggests that, if digital support is going to be successful, it is essential that the individual's life context, expectations, needs, and skills concerning what kind of support is given are taken into consideration.

Buddy does not require participants to engage with the materials on a daily basis. Rather, Buddy is designed as a low-intensity intervention, and the core component is an end-of-the-week prompt to self-assess lifestyle behaviors. Our results show that this design was not a suitable match for all participants. Students who perceived that they had not successfully changed in accordance with their expectations also expressed more dissatisfaction with the support received. For example, a loss of interest and feeling that they needed encouragement. Results from a study by Schroé et al.^45^ indicated that those who dropped out of a digital physical activity intervention received no additional value from the intervention. This could partially explain why some found that Buddy was not suitable to their circumstances and did not live up to their expectations. However, behavior change requires the capacity to translate information, reflections, and functions into action, which some students faced difficulties doing. For example, they experienced difficulties when formulating goals and could not self-manage healthy behaviors over time. Meanwhile students who felt that they could cope and deal with failures expressed confidence in their ability to change.

The four phenotypes identified by Burgermaster^48^ —which were called Activated, Inspired, Reinforced, and Indifferent—all responded differently to the school-based childhood obesity prevention intervention. They also had different psychosocial characteristics, with perceived risk and self-regulation differentiating the successful group.^48^ Those who were successful in making a change in the “Activated” phenotype are comparable to our “Compliant persona” identified in this study. While the not fully successful “Inspired” phenotype, who made attempts to adopt behavior change, is comparable to our “Kickstarter persona.”Meanwhile the “Reinforced” phenotype, which includes those who were already practicing healthy behaviors, aligns well with our “Personally developed persona.” Finally, the “Indifferent” phenotype, who was uninterested, relates well to our “Occupied persona.” It should be noted that we were unaware of this previous study throughout the analysis stage of our study, and so our personas were not developed with these phenotypes in mind. Nonetheless, the commonality across studies suggests that the personas we have identified may be suitable for use to describe individuals in other target populations beyond college and university students. We propose that, to ensure that the dissemination and implementation of digital interventions is effective, a variety of personas need to be considered when developing them. By doing so, we may avoid the development of interventions that match a singular conceptual image of the target population, such as “college and university students.” This may result in entirely different intervention designs for different individuals within the target population, with varying degrees of intensity and intervention materials.

However, no matter how much we refine, develop, and personalize digital interventions, it is unlikely that they will be a good fit for all individuals in the target population. Similarly, the utilization of digital interventions is not universal, with some finding them useful while others do not engage with them at all. To support “persona eight”—that is, those who did not find signing up for the Buddy trial appealing—we also need to develop interventions at a structural level. These structural level interventions include those that operate on societal and environmental levels,^56,57^ including the commercial determinants of health that shape norms among students.^58^ For instance, changing social structures and norms around alcohol consumption and tobacco use at universities, or providing students with affordable healthy food choices on campus. When we seek to understand students’ contexts using ecological models of health determinants, we visualize health as created in an interplay between the individual, the environment, and society. This illustrates how different layers of influence can threaten, promote, and protect health within the population.^59–61^ Furthermore, the understanding of students’ alcohol consumption views it as an organizing principle of university social life, including cultural conventions, expectations, and socially shared beliefs that alcohol is central to student life.^62^ Providing public health interventions in different delivery formats (face-to-face, digital, or in the community), using different activities at multiple levels of the ecological model (individual, group, or societal levels), appears to be the most effective and mutually reinforcing approach. Although it seems that in practice this is rarely implemented.^63^

### Strengths and limitations

This study focused on the qualitative aspect of students’ narratives at the start of, during, and after taking part in a trial of a behavior-change support intervention. Thus, the data represented what students recalled and remembered, including their experiences, perceptions, and interpretations, which implies a risk of recall bias. Additional data sources, such as repeated interviews and/or reflective diaries, could have helped participants keep track of their experiences. However, we opted to reduce participant burden considering that participants were already taking part in a trial which required data collection. Yet, experiences that have made an impact are often imprinted in the memory and may be retrieved and retold.^64^ A similar risk of bias is the social desire to meet others’ expectations,^65^ here presumably reduced by the large number of study participants (given the qualitative design). The use of telephone interviews enabled convenient participation by a large number of students at universities across Sweden, who were able to take part without putting additional demands on their time. Communication without being visible might also be preferable for some participants when dealing with sensitive topics, because it permits more anonymity and a reduced sense of social pressure and stigma.^66,67^ Furthermore, the design provided the study with empirical data in a real-life context from a varied sample of interview respondents, including both those who used the intervention and those who decided to stop using it.

Another limitation of the study is that the interview guide did not prompt respondents to discuss their personal or digital support preferences. Nevertheless, this was discussed by some of the respondents. For instance, the *Goal-oriented* persona acknowledged needing more hands-on support, while the *Personally developed* persona felt cared for despite being aware that Buddy was an automated program. We also did not investigate students’ screen behaviors or social media use in relation to their wellbeing, health, or studies, and respondents did not raise these aspects. The *Compliant persona* did, however, perceive Buddy as manageable alongside other commitments. Perhaps natural considering Buddy's low-intensity intervention format, requiring only a few seconds of reading text messages and not being linked to social media platforms.

Like all qualitative studies, the findings are not intended to be generalizable but rather to contribute to an understanding of how individuals interpret behavior change a posteriori. Nevertheless, the resulting personas may be applicable when targeting college and university students more generally and may even be transferable to young adults beyond the college and university population.

## Conclusions

The seven personas shaped from college and university students’ intentions and experiences of the behavior-change process embrace and illustrate the variability among the target group. At the outset, the students were approached as a relatively homogeneous group signing up to a digital intervention but shown to represent different experiences of the intervention. However, it was shown that the progress of behavior change depends on the interaction between the digital mode of delivery, the intervention materials of Buddy, the individual's expectations, needs, and skills, and their current life situation. This indicates that those designing lifestyle interventions should more often consider the end-users’ intentions, knowledge, and contexts. Although multiple interventions are likely to emerge, there may be a better fit with the target population.

## Supplemental Material

sj-doc-1-dhj-10.1177_20552076241245905 - Supplemental material for “Simply complicated”: Uncovering the processes of lifestyle behavior change among college and university students with access to a digital multiple lifestyle intervention Supplemental material, sj-doc-1-dhj-10.1177_20552076241245905 for “Simply complicated”: Uncovering the processes of lifestyle behavior change among college and university students with access to a digital multiple lifestyle intervention by Katarina Åsberg, Ann Catrine Eldh, Marie Löf and Marcus Bendtsen in DIGITAL HEALTH

sj-doc-2-dhj-10.1177_20552076241245905 - Supplemental material for “Simply complicated”: Uncovering the processes of lifestyle behavior change among college and university students with access to a digital multiple lifestyle intervention Supplemental material, sj-doc-2-dhj-10.1177_20552076241245905 for “Simply complicated”: Uncovering the processes of lifestyle behavior change among college and university students with access to a digital multiple lifestyle intervention by Katarina Åsberg, Ann Catrine Eldh, Marie Löf and Marcus Bendtsen in DIGITAL HEALTH

sj-doc-3-dhj-10.1177_20552076241245905 - Supplemental material for “Simply complicated”: Uncovering the processes of lifestyle behavior change among college and university students with access to a digital multiple lifestyle intervention Supplemental material, sj-doc-3-dhj-10.1177_20552076241245905 for “Simply complicated”: Uncovering the processes of lifestyle behavior change among college and university students with access to a digital multiple lifestyle intervention by Katarina Åsberg, Ann Catrine Eldh, Marie Löf and Marcus Bendtsen in DIGITAL HEALTH
