# Supplementary material for: “Simply complicated”: Uncovering the processes of lifestyle behavior change among college and university students with access to a digital multiple lifestyle intervention
Source: Digit Health. 2024 Apr 9;10:20552076241245905. doi: 10.1177/20552076241245905 (PMC11005484; doi:10.1177/20552076241245905)
Supplement: sj-doc-1-dhj-10.1177_20552076241245905 - Supplemental material for “Simply complicated”: Uncovering the processes of lifestyle behavior change among college and university students with access to a digital multiple lifestyle intervention [file sj-doc-1-dhj-10.1177_20552076241245905.doc]

## Appendix A - Description of the Buddy intervention

This appendix describes the development and the content of the Buddy intervention. The intervention was created with inspiration from the first four steps of the Intervention Mapping (IM) approach [1]. Buddy is a novel digital intervention, delivered via mobile phone, that targets multiple lifestyle behaviors (alcohol, diet, physical activity, and smoking) among university students in Sweden. Buddy is based on social cognitive models for behavior change, where environment, intentions, and skills often are highlighted as important for change [2,3]. Therefore, we identified and designed components which intended to affect these factors. In addition, the design of the components were based on previous research on digital lifestyle interventions conducted among university students in Sweden [4–11] and with the use of the BCTTv1 93-item taxonomy [12]. A full description of the Buddy intervention design is found in the study protocol [13]. The logic model in Figure 1 gives an overview of the reasoning behind the intervention, including outcomes and potential short-, mid- and long-term impact.


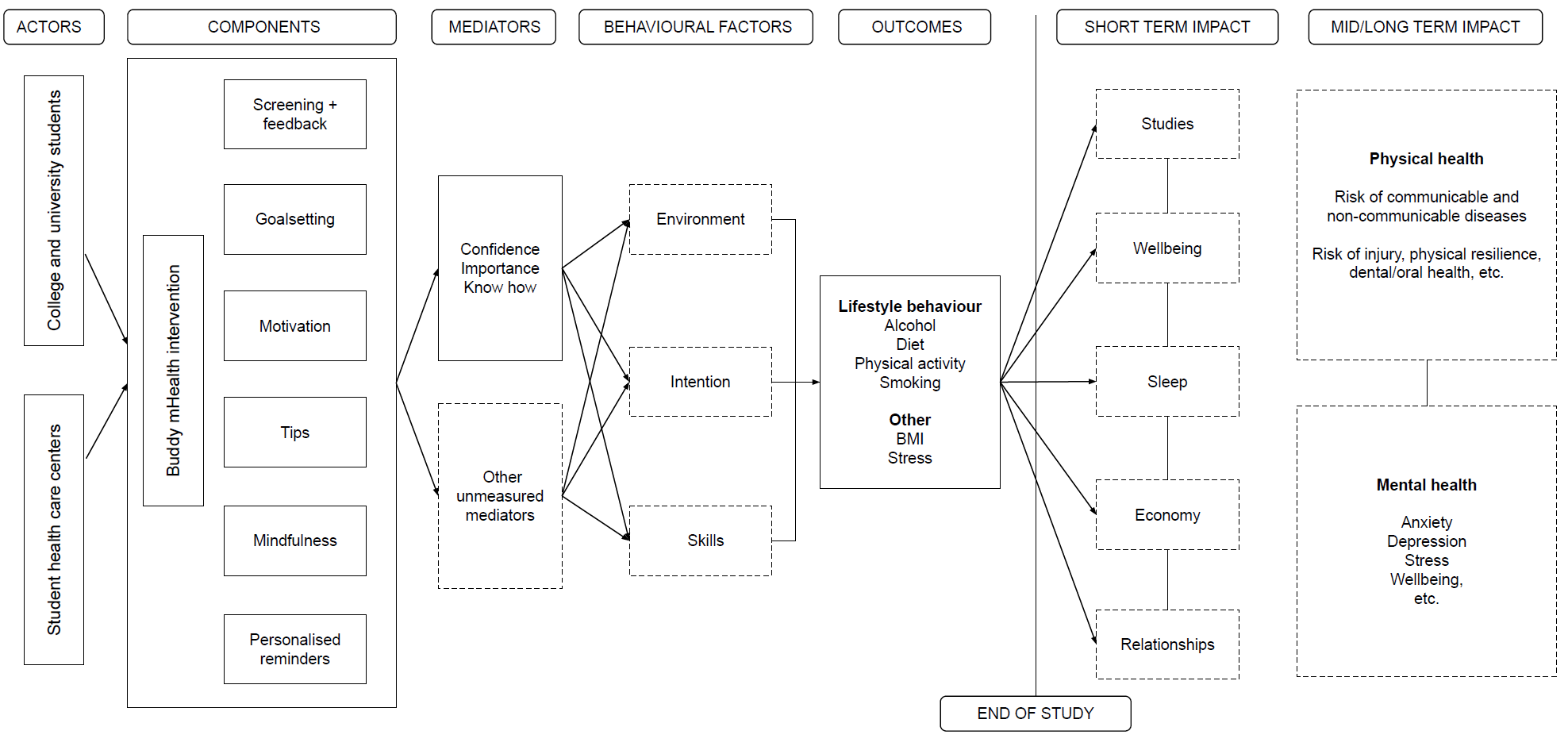


Figure 1. Logic model showing actors, intervention components, mediators, behavioral factors, outcomes, and short-, mid- and long-term impacts.

The intervention is intended to be used as a toolbox, enabling users to decide which intervention content they want to interact with. The intervention materials can be accessed at the participants’ discretion over a period of four months. Each Sunday afternoon, participants receive a text message with a link and a reminder to access Buddy.

The intervention consists of six components:

**1)** screening and feedback

**2)** goal-setting and planning

**3)** motivation

**4)** skills and know-how

**5)** mindfulness

**6)** self-authored text messages.

Screenshots of the intervention are found in Figure 1, showing: i) main menu, ii) screening questionnaire, and iii) feedback view.


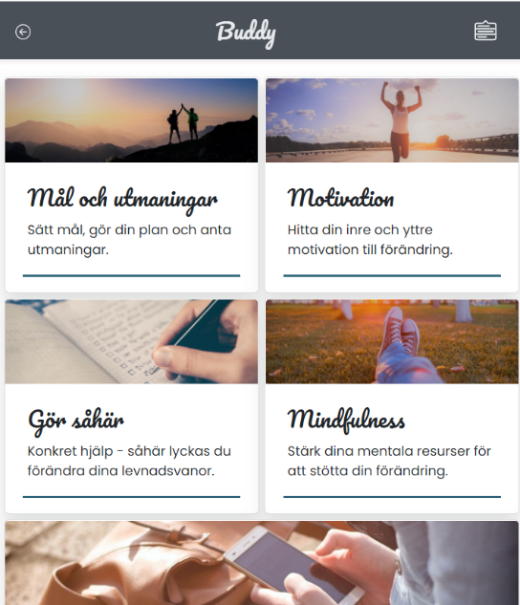


Figure 1 i) main menu


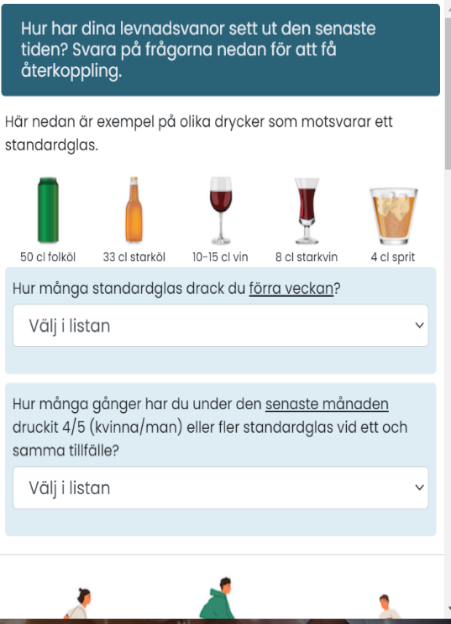

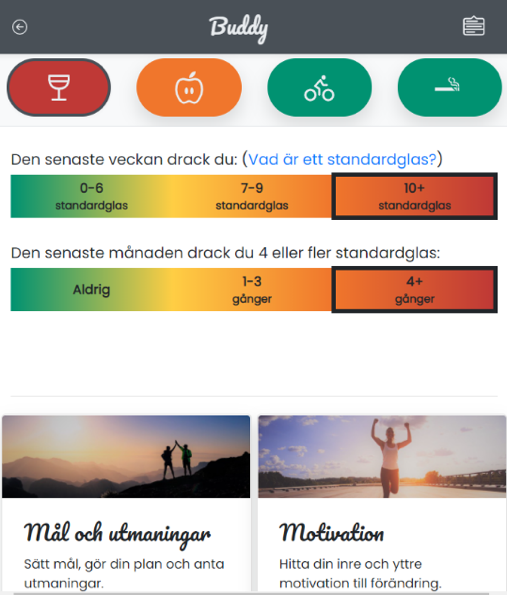


Figure 1 ii) Screening questionnaire and iii) feedback view

## Components

#### COMPONENT 1: Screening and feedback

The first component consists of screening and feedback. When tapping the link in the weekly text message, participants are asked to respond to a questionnaire regarding their current lifestyle behaviors, after which they are shown feedback on their current behavior in comparison with national guidelines. They are subsequently given access to the rest of the components appropriate to their randomized allocation. Self-monitoring has been shown to be a potentially effective strategy for reducing excessive alcohol consumption [9–12] and to promote healthy eating and physical activity [14,15]. When this component is absent, participants will not be asked to respond to the screening questionnaire but will instead be shown national guidelines without any feedback.

#### COMPONENT 2: Goal-setting and planning

The second component supports enhanced self-regulatory capacity and skills via goal-setting and planning. This includes setting goals for future behavior, preparing for triggers, and accepting both customized and ready-made challenges. Intervention content designed around goal-setting, action planning, practicing behavior, and habit formation, amongst other planning-related activities, have been shown to be important examples of effective lifestyle interventions [14,16–21]. Participants are reminded of the goals they have set, including any challenges they have accepted, via text message prompts throughout the week (up to four messages).

#### COMPONENT 3: Motivation

The third component aims to increase users’ awareness of their own motivation, encourage commitment, and boost motivation and self-efficacy. This is supported via texts, videos, and exercises relating to health, finances, and motivation awareness. Digital behavior-change interventions have been shown to have the capacity to increase self-efficacy; however, there is a lack of consensus across reviews as to which content works best to facilitate an increase in self-efficacy [22]. This component also allows participants to sign up for text messages containing motivational content to be sent to them throughout the week. Participants choose which behaviors they wish to receive messages for, with a maximum of 8–10 messages per week. The content of these messages has been derived from previously developed and evaluated interventions [4,5,23–27].

#### COMPONENT 4: Skills and know-how

The fourth component aims to increase users’ skills and know-how about how to make lasting behavioral changes. This includes concrete tips on how to initiate and maintain change in everyday life. For instance, participants are given strategies they can employ when going to parties where alcohol is served, or how to introduce vegetables into their breakfast. As with the third component, participants will be able to sign up for text messages with tips sent to them throughout the week (maximum 8–10 per week) – the content of which has also been derived from previously developed and evaluated interventions [4,5,23–27].

#### COMPONENT 5: Mindfulness

The fifth component aims to increase users’ awareness of their own lived experience and strengthen their capacity for a non-reactive, compassionate, and less stressful way of being in the world. The practices thus help participants to build the mental resources needed for behavior change. A set of mindfulness exercises, including guided meditations, are available in this component. These exercises are based on previous research, and are considered evidence-based methods to improve the mental well-being of clinical populations, while effects on behavior change in non-clinical settings are less well-studied [28–32].

#### COMPONENT 6: Self-authored text messages

The sixth component consists of self-composed text messages sent to participants throughout the week. Participants are allowed to author up to three messages to themselves and have them sent at specified intervals. For instance, a participant can write a message about their commitment to increasing their physical activity and decide to have it sent to them every Monday and Wednesday at 5pm. This type of activity seems generally under-studied in the literature, but has shown interesting preliminary results in an ongoing trial [33].

## References

1. Kay Bartholomew Eldredge L, Markham CM, Ruiter RAC, Fernández M, Kok G, Parcel GS. Planning health promotion programs: an intervention mapping approach. Fourth edi. San Francisco, CA: Jossey-Bass & Pfeiffer Imprints, Wiley; 2016.

2. Fishbein M, Triandis HC, Kanfer FH, Becker M, Middlestadt SE, Eichler A. Factors influencing behaviour and behaviour change. In: Handbook of Health Psychology. Psychology Press Taylor & Francis Group; 2001. p. 3–17.

3. Conner M, Norman P. Predicting Health Behavior: Research and Practice with Social Cognition Models. 2005.

4. Mussener U, Bendtsen M, Karlsson N, White IR, McCambridge J, Bendtsen P. Effectiveness of Short Message Service Text-Based Smoking Cessation Intervention Among University Students: A Randomized Clinical Trial. JAMA Intern Med. 2016 Mar;176(3):321–8.

5. Thomas K, Müssener U, Linderoth C, Karlsson N, Bendtsen P, Bendtsen M. Effectiveness of a text messaging–based intervention targeting alcohol consumption among university students: randomized controlled trial. JMIR mHealth uHealth. 2018;6(6):e146.

6. Mussener U, Bendtsen M, McCambridge J, Bendtsen P. User satisfaction with the structure and content of the NEXit intervention, a text messaging-based smoking cessation programme. BMC Public Health. 2016 Nov;16(1):1179.

7. Mussener U, Thomas K, Linderoth C, Leijon M, Bendtsen M. A Text Message-Based Intervention Targeting Alcohol Consumption Among University Students: User Satisfaction and Acceptability Study. JMIR Hum factors. 2018 Jul;5(3):e23.

8. Thomas K, Linderoth C, Bendtsen M, Bendtsen P, Mussener U. Text Message-Based Intervention Targeting Alcohol Consumption Among University Students: Findings From a Formative Development Study. JMIR mHealth uHealth. 2016 Oct;4(4):e119.

9. Bendtsen P, McCambridge J, Bendtsen M, Karlsson N, Nilsen P. Effectiveness of a proactive mail-based alcohol Internet intervention for university students: dismantling the assessment and feedback components in a randomized controlled trial. J Med Internet Res. 2012 Oct;14(5):e142.

10. McCambridge J, Bendtsen M, Karlsson N, White IR, Nilsen P, Bendtsen P. Alcohol assessment and feedback by email for university students: main findings from a randomised controlled trial. Br J Psychiatry. 2013 Nov;203(5):334–40.

11. Bendtsen P, Bendtsen M, Karlsson N, White IR, McCambridge J. Online Alcohol Assessment and Feedback for Hazardous and Harmful Drinkers: Findings From the AMADEUS-2 Randomized Controlled Trial of Routine Practice in Swedish Universities. J Med Internet Res. 2015 Jul;17(7):e170.

12. Michie S, Whittington C, Hamoudi Z, Zarnani F, Tober G, West R. Identification of behaviour change techniques to reduce excessive alcohol consumption. Addiction. 2012 Aug;107(8):1431–40.

13. Åsberg K, Lundgren O, Henriksson H, Henriksson P, Bendtsen P, Löf M, et al. Multiple lifestyle behaviour mHealth intervention targeting Swedish college and university students: protocol for the Buddy randomised factorial trial. Vol. 11, BMJ Open. 2021.

14. Michie S, Abraham C, Whittington C, Mcateer J. Effective Techniques in Healthy Eating and Physical Activity Interventions : A Meta-Regression. 2009;28(6):690–701.

15. Murray JM, Brennan SF, French DP, Patterson CC, Kee F, Hunter RF. Effectiveness of physical activity interventions in achieving behaviour change maintenance in young and middle aged adults: A systematic review and meta-analysis. Soc Sci Med. 2017 Nov;192:125–33.

16. Knittle K, Nurmi J, Crutzen R, Hankonen N, Beattie M, Dombrowski SU. How can interventions increase motivation for physical activity? A systematic review and meta-analysis. Health Psychol Rev. 2018 Sep;12(3):211–30.

17. Howlett N, Trivedi D, Troop NA, Chater AM. Are physical activity interventions for healthy inactive adults effective in promoting behavior change and maintenance, and which behavior change techniques are effective? A systematic review and meta-analysis. Transl Behav Med. 2019 Jan;9(1):147–57.

18. Ashton LM, Sharkey T, Whatnall MC, Williams RL, Bezzina A, Aguiar EJ, et al. Effectiveness of Interventions and Behaviour Change Techniques for Improving Dietary Intake in Young Adults: A Systematic Review and Meta-Analysis of RCTs. Nutrients. 2019 Apr;11(4).

19. Garnett C V, Crane D, Brown J, Kaner EFS, Beyer FR, Muirhead CR, et al. Behavior Change Techniques Used in Digital Behavior Change Interventions to Reduce Excessive Alcohol Consumption: A Meta-regression. Ann Behav Med. 2018 May;52(6):530–43.

20. McCrabb S, Baker AL, Attia J, Skelton E, Twyman L, Palazzi K, et al. Internet-Based Programs Incorporating Behavior Change Techniques Are Associated With Increased Smoking Cessation in the General Population: A Systematic Review and Meta-analysis. Ann Behav Med. 2019 Feb;53(2):180–95.

21. Ek A, Alexandrou C, Söderström E, Bergman P, Delisle Nyström C, Direito A, et al. Effectiveness of a 3-Month Mobile Phone-Based Behavior Change Program on Active Transportation and Physical Activity in Adults: Randomized Controlled Trial. JMIR mHealth uHealth. 2020 Jun;8(6):e18531.

22. Newby K, Teah G, Cooke R, Li X, Brown K, Salisbury-Finch B, et al. Do automated digital health behaviour change interventions have a positive effect on self-efficacy? A systematic review and meta-analysis. Health Psychol Rev. 2020 Jan;1–19.

23. Mussener U, Bendtsen M, Karlsson N, White IR, McCambridge J, Bendtsen P. SMS-based smoking cessation intervention among university students: study protocol for a randomised controlled trial (NEXit trial). Trials. 2015 Apr;16:140.

24. Thomas K, Bendtsen M, Linderoth C, Karlsson N, Bendtsen P, Mussener U. Short message service (SMS)-based intervention targeting alcohol consumption among university students: study protocol of a randomized controlled trial. Trials. 2017 Apr;18(1):156.

25. Müssener U, Bendtsen M, McCambridge J, Bendtsen P. User satisfaction with the structure and content of the NEXit intervention, a text messaging-based smoking cessation programme. BMC Public Health. 2016;16(1):1179.

26. Mussener U, Thomas K, Linderoth C, Leijon M, Bendtsen M. A Text Message-Based Intervention Targeting Alcohol Consumption Among University Students: User Satisfaction and Acceptability Study. JMIR Hum factors. 2018;5(3):e23.

27. Thomas K, Linderoth C, Bendtsen M, Bendtsen P, Müssener U. Text Message-Based Intervention Targeting Alcohol Consumption Among University Students: Findings From a Formative Development Study. JMIR mHealth uHealth. 2016;4(4):e119.

28. Crane RS, Brewer J, Feldman C, Kabat-Zinn J, Santorelli S, Williams JMG, et al. What defines mindfulness-based programs? The warp and the weft. Psychol Med. 2017 Apr;47(6):990–9.

29. Creswell JD. Mindfulness Interventions. Annu Rev Psychol. 2017 Jan;68(1):491–516.

30. Wong SYS, Chan JYC, Zhang D, Lee EKP, Tsoi KKF. The Safety of Mindfulness-Based Interventions: a Systematic Review of Randomized Controlled Trials. Mindfulness (N Y). 2018 Oct;9(5):1344–57.

31. Galante J, Friedrich C, Dawson AF, Modrego-Alarcón M, Gebbing P, Delgado-Suárez I, et al. Mindfulness-based programmes for mental health promotion in adults in nonclinical settings: A systematic review and meta-analysis of randomised controlled trials. Patel V, editor. PLOS Med. 2021 Jan;18(1):e1003481.

32. Neff KD, Germer CK. A Pilot Study and Randomized Controlled Trial of the Mindful Self-Compassion Program. J Clin Psychol. 2013 Jan;69(1):28–44.

33. Bendtsen M, McCambridge J. Reducing Alcohol Consumption Among Risky Drinkers in the General Population of Sweden Using an Interactive Mobile Health Intervention: Protocol for a Randomized Controlled Trial. JMIR Res Protoc. 2019;8(4):e13119.
