# Supplementary material for: “Simply complicated”: Uncovering the processes of lifestyle behavior change among college and university students with access to a digital multiple lifestyle intervention
Source: Digit Health. 2024 Apr 9;10:20552076241245905. doi: 10.1177/20552076241245905 (PMC11005484; doi:10.1177/20552076241245905)
Supplement: sj-doc-3-dhj-10.1177_20552076241245905 - Supplemental material for “Simply complicated”: Uncovering the processes of lifestyle behavior change among college and university students with access to a digital multiple lifestyle intervention [file sj-doc-3-dhj-10.1177_20552076241245905.doc]

# Interview guide (following introductions)

1. As you may recall from when you signed up four months ago, that the Buddy study is about lifestyle and lifestyle behaviors… It should have been [insert month] then? (…) If you think of when you first heard about the study…**What** did you think of **then**, with regards to the study?

2. Then you had access to a support tool in your mobile phone…Tell me, what happened **then**, in the beginning? [probes: intention(s); ambition(s); expectation(s); goal(s); plan(s)]

3. If you think about what happened **next**, considering your lifestyle behaviors? Did you make any changes?
[If responding positive] Please tell me (more)… What did you change; how did it work?

[If responding negative] Tell me, what happened?

[probes: what kind of change OR why not; multiple/linked; other kind of support; hard/easy; how; examples]

4. Can I now ask you to **further** consider changing lifestyle behaviors, it can be big or small: what do you think can prevent one from changing, or enable one to make changes in one’s lifestyle behaviors? (…) What’s your experience? (…) What reactions, strategies, and experiences would you say that you have had yourself?

5. Now I’d like to ask you to grab your mobile phone. Please do not activate or use it, but just hold it in your hand like you would do if you were about to use it (…) OK, so **now**, I’d like you to imagine that you were about to access Buddy. (…) Please tell me what you think of Buddy as a potential tool for making a change in lifestyle behaviors?... You may access the support if you want (…) Any (further) thoughts on how Buddy can be such a tool, to change lifestyle behaviours?

[probes: positive/negative – technicalities – user friendliness – areas of relevance – support/drawback]

6. Thank you, these were the queries I had… But I´d like to know if there’s anything you think of now?

[probes: it may be things we’ve talked about, or something that hasn’t come up]

*The interview was concluded with a thank you, and a reminder to contact the interviewer if any further aspects came to mind, (including adding, amending, or withdrawing details).*

NOTES: … indicates short pause, and (…) a slightly longer silence. Words emphasized in text were emphasized in phrasing for each interview.
